# Vaccine-Induced Immune Thrombotic Thrombocytopenia (VITT)-like Syndrome: A Case Report and Some Considerations on a Novel Diagnostic and Therapeutic Challenge

**DOI:** 10.3390/diagnostics16020257

**Published:** 2026-01-13

**Authors:** Lorenzo Delfino, Sara Moruzzi, Michela Carrillo, Silvia Suardi, Sabrina Genesini, Linda Schönborn, Jan Wesche, Giulia Salandini, Carlotta Spillere, Gabriele Costa, Salvatore Simari, Francesca Pizzolo, Enrico Polati, Giancarlo Mansueto, Giorgio Gandini, Simonetta Friso, Thomas Thiele, Nicola Martinelli

**Affiliations:** 1 Department of Medicine, University of Verona, 37129 Verona, Italy; lorenzo.delfino@aovr.veneto.it (L.D.); sara.moruzzi@aovr.veneto.it (S.M.); michela.carrillo@outlook.com (M.C.); silvia.suardi@aovr.veneto.it (S.S.); carlotta.spillere94@gmail.com (C.S.); gabriele.costa@studenti.univr.it (G.C.); francesca.pizzolo@univr.it (F.P.); simonetta.friso@univr.it (S.F.); 2 Transfusion Medicine Department, Integrated University Hospital of Verona, 37129 Verona, Italy; sabrina.genesini@aovr.veneto.it (S.G.); giorgio.gandini@aovr.veneto.it (G.G.); 3 Institut für Transfusionsmedizin, Universitätsmedizin Greifswald, 17487 Greifswald, Germany; linda.schoenborn@med.uni-greifswald.de (L.S.); jan.wesche@med.uni-greifswald.de (J.W.); thomas.thiele@med.uni-greifswald.de (T.T.); 4 Department of Radiology, University of Verona, 37129 Verona, Italy; giulia.salandini@aovr.veneto.it (G.S.); giancarlo.mansueto@univr.it (G.M.); 5 Department of Surgery, Section of Anaesthesia, University of Verona, 37129 Verona, Italy; salvatore.simari@aovr.veneto.it (S.S.); enrico.polati@univr.it (E.P.)

**Keywords:** platelet-activating anti-PF4 disorders, VITT-like syndrome, venous thromboembolism, immunothrombosis

## Abstract

**Background and Clinical Significance:** Disorders caused by platelet-activating antibodies targeting platelet factor 4 (PF4) are recognized as the cause of severe thrombotic events and are not restricted to heparin-induced thrombocytopenia (HIT). **Case Presentation:** We report a 67-year-old man with thrombocytopenia and extensive portal-splenic-mesenteric vein thrombosis complicated by intestinal ischemia. Despite intravenous unfractionated heparin (UFH), his condition worsened toward pulmonary embolism, septic shock, and multi-organ failure. Thrombolysis with alteplase was also ineffective. Both thrombophilia testing and autoimmune panels were negative, including those for antiphospholipid syndrome. An anti-PF4 immune thrombotic disorder was hypothesized. Therefore, argatroban was initiated instead of UFH therapy and intravenous immune globulin (IVIG) was administered. The platelet count increased and the patient’s clinical condition progressively improved. An anti-PF4/heparin assay on a blood sample collected before IVIG was highly positive. Platelet activation assays did not demonstrate an increased activation after the addition of heparin (the Heparin-Induced Platelet Activation [HIPA] assay was negative) though increased activation was observed with the addition of PF4 (the PF4-Induced Platelet Activation [PIPA] assay was positive), thus defining a VITT-like syndrome. **Conclusions:** This case report highlights the crucial function of having adequate laboratory facilities available to disentangle different anti-PF4 disorders for an accurate definition of a specific diagnosis, such as VITT-like syndrome, thereby allowing for the most appropriate therapeutic management of these complex pathological conditions. The clinical suspicion of an anti-PF4 immune disorder should be considered in cases of severe, otherwise unexplained, thrombotic events associated with thrombocytopenia. Specific tests like HIPA and PIPA are essential for definitive diagnosis.

## 1. Introduction

Vaccine-induced immune thrombotic thrombocytopenia (VITT)-like syndrome represents the latest condition added to the growing and heterogeneous family of platelet-activating anti-platelet factor 4 (PF4) disorders, the history of which began more than 60 years ago with the first description of probable cases of heparin-induced thrombocytopenia (HIT) [1]. During the 1970s the immunologic basis for HIT was suggested by studies reporting evidence of platelet activation induced by serum and purified immunoglobulins of patients with HIT [2]. In 1992 antibodies recognizing complexes of PF4/heparin were identified as the cause of HIT [3]. A notable characteristic of the anti-PF4/heparin immune response is that a considerable proportion of patients exposed to heparin develop non-pathogenic (anti-PF4 type 1) antibodies. Although the clinical diagnosis of HIT can be achieved by combining clinical scores, like 4Ts, and anti-PF4/heparin immunoassay results, definitive “molecular” diagnosis of HIT requires the identification of the much less frequent heparin-dependent, platelet activating (anti-PF4 type 2) antibodies [4,5].

However, the traditional view of HIT as exclusively heparin-dependent has been questioned over the last two decades as it came to be recognized that some patients may also exhibit atypical characteristics, where clinical and laboratory manifestations began or persisted after heparin discontinuation (autoimmune HIT), began after exposure to very low doses of heparin (heparin “flush” HIT), or appeared even in the absence of a positive history for heparin exposure (spontaneous HIT, which was originally observed in some specific clinical settings, like after knee replacement surgery or after viral infection) [6,7].

Most recently, during the COVID-19 pandemic, the term VITT was introduced to describe a very rare syndrome characterized by thrombocytopenia and severe prothrombotic diathesis following the administration of the two adenovirus vector-based SARS-CoV-2 vaccines, ChAdOx1nCoV-19 or Ad26.COV2.S [8,9,10]. VITT patients were usually healthy subjects without known prothrombotic risk factors who often developed unusual-site venous thromboembolism, such as cerebral venous thrombosis and splanchnic vein thrombosis [11,12,13]. It is noteworthy that patients with VITT required specific therapeutic management that requires anticoagulation associated with a high-dose of intravenous immunoglobulin (IVIG) to reduce platelet activation mediated by the interaction between anti-PF4 antibodies and the FcyRIIa receptor expressed on the platelet surface [14].

HIT and VITT antibodies can be differentiated by their binding to distinct PF4 epitopes. HIT antibodies bind to PF4/heparin complexes (anti-PF4 type 2), while VITT antibodies target PF4 in the absence of heparin by attaching to the heparin-binding region on PF4 and are heparin-independent, platelet activating (anti-PF4 type 3) antibodies [15]. Anti-PF4 type 3 antibodies cause thromboinflammation by binding to PF4 and are the main pathophysiological molecular basis underlying VITT [7]. Anti-PF4 type 3 antibodies have therefore a very high sensitivity and specificity for diagnosis of VITT [16] but have also been identified in autoimmune and spontaneous HIT, in such cases together with anti-PF4 type 2 antibodies [6]. Notably, as it refers to platelet activation assays, the PF4-Induced Platelet Activation (PIPA) assay is more sensitive than the Heparin-Induced Platelet Activation (HIPA) assay for detecting VITT antibodies [6,16,17].

The discovery of VITT paved the way for a growing interest in platelet activating disorders and their pathogenetic mechanisms. Clinical syndromes resembling the characteristics of VITT (with both thrombosis and thrombocytopenia, as well as with negative HIPA and positive PIPA tests at laboratory assessment) have been described in subjects without clear evidence of recent exposure to either heparin or adenovirus vector-based SARS-CoV-2 vaccines, thus adding the group of VITT-like syndromes to the wide panorama of platelet-activating anti-PF4 disorders. It is noteworthy that many VITT-like syndromes appear to be related to previous adenovirus infections [17,18,19,20,21,22]. However, VITT-like syndromes are not only limited to adenoviruses infection and have also been described after viral infections by respiratory syncytial virus (RSV) or cytomegalovirus (CMV), as well as following vaccination for human papillomavirus (HPV) [17,23,24,25]. Moreover, a case of neonatal VITT-like syndrome caused by maternal transfer of anti-PF4 antibodies has been recently reported by Häusler et al. [26]. Studies on the molecular pathways underlying the mechanisms of thrombosis in anti-PF4 disorders have demonstrated that PF4 can form stable complexes with adenoviruses through electrostatic interaction. Remarkably, these conformational changes in PF4 induced by electrostatic binding can lead to the formation of neoepitope and the subsequent development of anti-PF4 antibodies [27,28]. However, it is important to note that we still do not know which antigens trigger the anti-PF4 immune response or why immune tolerance can be disrupted following vaccination or viral infections, leading to the formation of pathological anti-PF4 type 3 antibodies.

Finally, a few cases characterized by a history of recurrent thrombosis and the chronic persistence of anti-PF4 antibodies have been reported among subjects with monoclonal gammopathy of unknown significance (MGUS), thus defining the new concept of monoclonal gammopathy of thrombotic significance (MGTS) [29,30,31]. In these cases the monoclonal paraprotein plays a key role in the pathogenesis of the prothrombotic diathesis by causing platelet activation through the binding to PF4 in a heparin-independent manner. Despite the serological features resembling a VITT-like profile, these anti-PF4 antibodies are characterized by significative differences from those found in acute VITT, specifically related to the target epitope. More precisely, anti-PF4 antibodies in MGTS have unique amino acid sequences in their binding epitopes, but they share similar negatively charged antigen-binding regions (paratopes), distinguishing them from other anti-PF4 disorders [32,33]. From a therapeutic perspective, it is intriguing to report how oncohematological treatments, such as Bruton’s tyrosine kinase (BTK) inhibitors or even chemotherapy most commonly used for myeloma, can provide substantial benefits for patients with MGTS, especially if conventional therapy with anticoagulant and/or antiplatelet drugs proves insufficiently effective [34,35].

Therefore, platelet-activating anti-PF4 disorders have opened and are yet opening new diagnostic and therapeutic strategies for the management of thrombotic diseases. However, anti-PF4 disorders remain poorly understood and likely underdiagnosed in real clinical practice because of the difficulties of a proper laboratory diagnosis (most laboratories are not adequately equipped for platelet activation functional assays like HIPA and PIPA) and the wide and somewhat blurred boundaries of anti-PF4 disorders, from classical to spontaneous HIT, from VITT to VITT-like syndrome. Finally, the diagnostic challenge is still evolving and further complicated by the observation that additional antigens leading to platelet activating immune complexes may also exist [36].

The following case report of VITT-like syndrome highlights the critical role of clinical suspicion for a prompt initiation of differential diagnosis and therapeutic management for disorders associated with platelet-activating antibodies targeting PF4. It also shows that accurate diagnostic confirmation depends upon specialized and appropriate laboratory evaluation.

## 2. Case Presentation

A 67-year-old man was admitted to the Emergency Department (ED) of our hospital due to acute and severe abdominal pain. Two weeks before the onset of symptoms, he experienced flu-like symptoms and underwent a short course of antibiotic therapy with cefixime. His medical history included arterial hypertension (treated with ramipril), benign prostatic hyperplasia (treated with tamsulosin), and asymptomatic chronic hepatitis B virus (HBV) infection. He had received the SARS-CoV-2 vaccination (adenovirus vector-based vaccine—ChAdOx1nCoV-19) two years prior and had not receive subsequent vaccinations. Neither personal nor family history of venous thromboembolism was reported.

**Figure 1:**
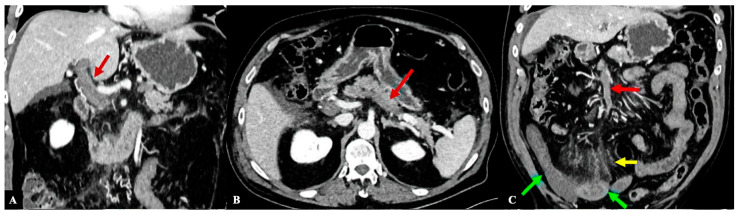
Panels (**A**–**C**) show massive spleno-mesenteric-portal thrombosis and intestinal ischemia by computed tomography (CT) imaging at time of hospital admission. (**A**) Coronal CT scan in the post-contrast venous phase showing complete thrombosis of the portal vein (red arrow). (**B**) Axial CT scan in the post-contrast venous phase showing complete thrombosis of the splenic vein (red arrow). (**C**) Coronal CT scan in the post-contrast venous phase showing complete thrombosis of the superior mesenteric vein (red arrow). The intestinal loops, especially on the right side, showed thickening and hypoperfusion of the walls with trilaminar appearance (“target’’ sign—indicated by green arrows) and edema of mesenteric adipose tissue (yellow arrow).

Blood examination at ED showed mild thrombocytopenia (94,000/mcL), high white blood cell count (19,330/mcL), and very high levels of D-dimer (123,062 mcg/L). Chest X-ray did not show infiltrates suggestive for pneumonia, and the nasopharyngeal swab for SARS-CoV-2 was negative. An abdomen computed tomography scan showed a massive splanchnic (portal-splenic-mesenteric) vein thrombosis causing widespread intestinal ischemia (Figure 1A–C). Anticoagulant therapy with intravenous unfractionated heparin (UFH) was started and the patient was admitted to the Intensive Care Unit.

Despite the appropriate UFH therapy within the adequate therapeutic range, the patient’s condition deteriorated—the patient also developed pulmonary embolism without evidence of deep vein thrombosis of the lower limbs and the intestinal ischemia was complicated by septic shock and multi-organ failure. Empirical antibiotic therapy and haemodynamic support were initiated. Given the progression of thrombosis, systemic thrombolysis with alteplase was administered. Despite the thrombolytic treatment, there was no clinical improvement and the extent of the thrombosis remained largely unchanged at imaging control.

Anti-cardiolipin and anti-beta2-glycoprotein1 antibodies were negative, and factor V Leiden, prothrombin 20210G>A, and JAK2 mutations were absent. Autoimmunity screening was negative, and the clone of paroxysmal nocturnal haemoglobinuria was not detected. Serum protein electrophoresis did not show qualitative and quantitative alterations.

Considering the lack of response to UFH therapy, the presence of mild thrombocytopenia at the onset of clinical manifestations, and the history of a recent flu-like infection, an anti-PF4 immune thrombotic disorder was supposed. More precisely, the clinical suspicion was for an episode of spontaneous HIT. An anti-PF4/heparin enzyme immunoassay was performed and was strongly positive. Therefore, it was recommended to start argatroban instead of UFH anticoagulant therapy and to administer a cycle of IVIG at 0.4 g/kg/day for a total of 5 days.

**Figure 2:**
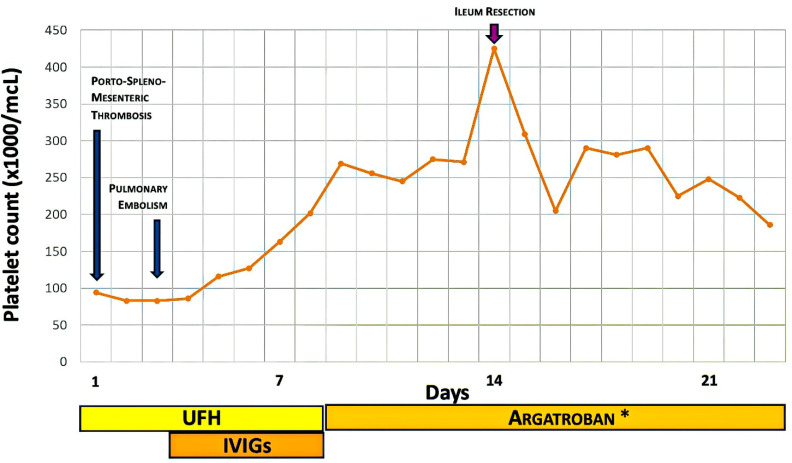
Platelet count during the first weeks of hospitalization: the platelet count was low for four days, then rose immediately after IVIG treatment and before starting argatroban therapy. On day fourteen, it dropped sharply following ileal resection surgery but returned to normal a few days post-surgery. IVIG: intravenous immune globulin; UFH: unfractionated heparin. * The switch to argatroban in place of UFH therapy was performed with 5 days of delay relative to the indication to start it together with the IVIG cycle.

Despite a 5-day delay in starting argatroban therapy, the platelet count increased immediately with the IVIG treatment (Figure 2), and remained stable within normal ranges.

Although the patient underwent ileum surgical resection due to bowel ischemia and had severe renal insufficiency, which required short-term haemodialysis, the overall clinical condition improved gradually. Finally, the anticoagulant treatment was switched from argatroban to warfarin (at discharge, renal function remained impaired with an estimated glomerular filtration rate of approximately 15 mL/min).

**Figure 3:**
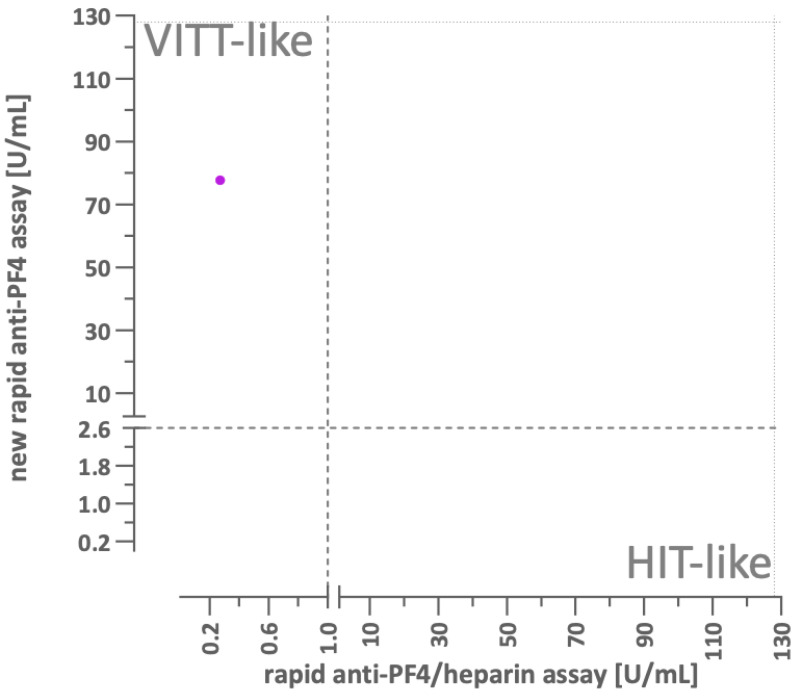
Platelet activation induced by the patient’s antibodies was evaluated using a washed platelet assay in the presence of heparin (HIPA) or PF4 (PIPA). The x-axis represents the functional assay results following heparin exposure (cutoff value represented by the dashed vertical line), while the y-axis displays results after PF4 exposure (cutoff value represented by the dashed horizontal line). The patient’s sample (purple dot) demonstrated positivity exclusively in the PF4-dependent functional assay, which is characteristic of VITT-like antibodies. Details on the assay are provided in reference [17].

Subsequently, on the same blood sample originally tested with the anti-PF4/heparin immunoassay, platelet activation assays revealed no increased activation upon the addition of heparin (HIPA assays). However, platelet activation instantly occurred with the addition of PF4 alone (PIPA assays). In addition, IgG antibodies recognizing PF4 only but not anti-PF4/heparin complexes were confirmed in an experimental chemiluminescent assay [17] (Figure 3). Therefore, the initial hypothesis of spontaneous HIT has been dismissed, while the diagnosis of VITT-like syndrome was proposed.

**Figure 4:**
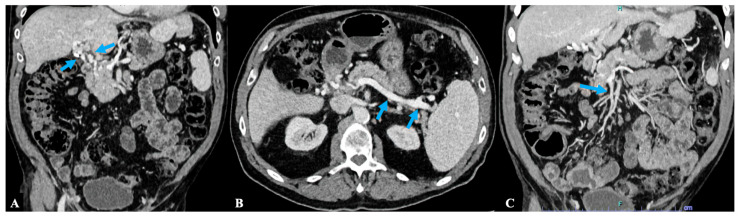
Panels (**A**–**C**) show evolution of spleno-mesenteric-portal thrombosis by computed tomography (CT) imaging after 1 year of follow-up, displaying the complete recanalization of previous portal-splenic-mesenteric vein thrombosis with the development of some collateral vein networks. (**A**) Coronal CT scan in the post-contrast venous phase showing recanalization of the portal circulation through the formation of collateral circles-portal cavernoma (blue arrows). (**B**) Axial CT scan in the post-contrast venous phase showing complete recanalization of the splenic vein (blue arrows). (**C**) Coronal CT scan in the post-contrast venous phase showing recanalization of the superior mesenteric vein (blue arrow).

After about 1 year of follow-up the anti-PF4/heparin assay was negative. Computed tomography imaging of the abdomen showed the complete recanalization of previous portal-splenic-mesenteric vein thrombosis with the development of some collateral vein networks (Figure 4A–C). The patient was clinically stable and substantially asymptomatic. Renal function improved and anticoagulant therapy was switched from warfarin to low-dose apixaban for the prevention of thrombotic recurrence.

## 3. Discussion

Platelet-activating anti-PF4 antibodies are the underlying cause of the many immune-mediated prothrombotic disorders and can be considered a paradigmatic example of immunothrombosis. In 2012, Engelmann and Massberg introduced the concept of immunothrombosis, describing thrombosis as a normal process that is crucial to the immune system’s defence against pathogens. Such processes involve complex interactions between molecules and cells from both the immune system and haemostasis. This intricate network operates in the limited space of microvessels and is thought to help keep potential pathogens confined, thus making it easier for the immune system effectors to identify and eliminate them [37]. Immunothrombosis, being a localized process, generally does not result in significant disruptions to organ perfusion. Nevertheless, an abnormal and uncontrolled activation of immunothrombosis can be detrimental to the host, promoting a disproportionate state of hypercoagulability which is often accompanied by concurrent activation of platelets, monocytes, neutrophils, and endothelial cells, thereby fostering thromboinflammation that eventually facilitates the propagation of thrombosis, and ultimately leads to organ damage [38,39,40,41,42]. From this point of view, it is worthy of note that anti-PF4 antibodies can trigger thrombosis also beyond Fcγ receptor IIA (FcγRIIA)-dependent platelet activation. Anti-PF4 antibodies have been shown to play a role in activating neutrophils and the consequent release of neutrophil extracellular traps (NETs) has been proposed as a major driver of prothrombotic diathesis in HIT [43].

Importantly, it is now understood that other factors beyond just heparin exposure or SARS-CoV-2 vaccination can also act as triggers for anti-PF4 antibodies. Other different antigens can lead to the formation of platelet activating immune complexes [36]. Müller and coauthors recently compiled a comprehensive review on the heterogeneous conditions associated with HIT-like or VITT-like disorders [44]. Müller et al., concomitantly, highlighted the importance of a prompt recognition and detailed characterization of underlying anti-PF4 disorders in patients presenting with thrombocytopenia and thrombosis, since precisely those steps are essential for an effective therapeutic management [44]. It is noteworthy that, in the case of anti-PF4 disorders, only certain specific types of pathogenic anti-PF4 antibodies—such as anti-PF4 type 2 (HIT-related) and/or type 3 (VITT-related) antibodies—lead to immunothrombosis. On the other hand, non-pathogenic antibodies (anti-PF4 type 1) have been identified in a relevant proportion of individuals exposed to heparin, as well as among healthy blood donors and approximately 5% to 8% of the general population following SARS-CoV-2 vaccination [45,46]. Therefore, the clinical picture within which anti-PF4 antibodies are searched for and possibly identified is crucial for an adequate interpretation of their biological and clinical significance.

In the clinical case described in the present report, the positive result of the immunoassay for anti-PF4 antibodies was associated with clinical manifestations strongly suggestive for a pathological anti-PF4 response with both unexplained thrombocytopenia and severe thrombotic complications. Our initial clinical suspicion was for spontaneous HIT. Therefore, we started both immunomodulatory therapy with IVIG and non-heparinic anticoagulant therapy with argatroban.

However, anti-PF4 antibodies detected by standard immunoassays are unable to distinguish between the various subsets of anti-PF4 antibodies and, therefore, cannot accurately classify the specific type of anti-PF4 disorder. This presents significant diagnostic implications, as functional assays capable of identifying and characterizing platelet-activating anti-PF4 antibodies—such as anti-PF4 type 2 (HIT-related) and/or type 3 (VITT-related) antibodies—are available only in a limited number of specialized laboratories [47,48].

The distinction between HIT or VITT antibody profiles could not be performed at our hospital and was achieved only later in a specialized reference laboratory on frozen serum samples, finally leading to the diagnosis of VITT-like syndrome. This issue emphasizes how laboratory facilities and adequate assays are crucial to disentangling the different anti-PF4 disorders and to making possible the accurate definition of a specific diagnosis, such as VITT-like syndrome. The availability of and collaborative networking among these laboratory services represent the decisive diagnostic challenge in order to be able to disseminate in clinical practice the knowledge, the recognition, and the treatment of anti-PF4 disorders.

VITT-like syndromes could be defined as novel thrombotic thrombocytopenic entities associated with anti-PF4 antibodies but independent from heparin or adenovirus vaccine administration [36]. As regards the etiopathogenesis of the VITT-like syndrome in our patient, we speculate that the disease had been sparked by the previous infectious episode. Earlier observations indicate that symptomatic human adenovirus infection can trigger VITT-like syndrome [18]. Adenoviruses are a common cause of respiratory illnesses and can manifest as a flu-like syndrome [49], as that reported by the patient before the outbreak of thrombotic events. Furthermore, since the patient’s serum protein electrophoresis was normal, MGTS can be reasonably excluded. However, we recognize as a substantial limitation of this report the fact we were unable to investigate adequately by either serology or viral DNA detection this etiopathogenetic hypothesis.

From a therapeutic point of view, the treatment of VITT-like syndromes derives from the experience of classic VITT and is based on anticoagulation, preferably with non-heparin anticoagulants, since a cross-reactivity with PF4/heparin complexes cannot be excluded [5,50,51,52,53]. But anticoagulation is not the only treatment. It is well known how immune complexes against PF4 interact with FcɣRIIa expressed on cellular surface, generating intracellular signals and finally leading to activation of platelets as well as monocytes and neutrophil granulocytes. Modulating FcγRIIA receptor activation with IVIG is particularly important. High-dose IVIG can rapidly block the interaction between anti-PF4 antibodies and the FcγRIIA receptor, thereby reducing platelet activation and improving platelet counts [44]. For the future, notably, other immunomodulatory drugs, like the BTK inhibitor ibrutinib, targeting the anti-PF4 antibody-mediated activation pathways downstream of FcγRIIA, have been proposed as additional therapy or already used as rescue therapy in anti-PF4 disorders [54,55].

As for therapeutic management, it is also worthy of note that our patient’s platelet count increased progressively with IVIG alone, even though the treatment with argatroban was started late. This delay did not seem to be linked to any worsening or complications of the disease, thereby suggesting that immunomodulation, rather than the type of anticoagulant drugs, is the cornerstone of the therapeutic management of VITT-like syndrome. This response aligns with the nature of VITT antibodies, which target a PF4 epitope different from HIT heparin-dependent sites, thus resulting in a predominantly heparin-independent platelet-activating disorder [44]. Remarkably, after about 1 year of follow-up the anti-PF4 assay was negative in our patient. Based on these findings, as well as evidence that heparin can inhibit VITT antibody-mediated platelet activation [56], it may be inferred that heparin therapies are not absolutely contraindicated in this case if clinically indicated, in contrast to individuals with a confirmed history of classical HIT [57].

## 4. Conclusions

Anti-PF4 disorders have recently opened up novel horizons for a better understanding of immunothrombosis, and for an improved, potentially life-saving, therapeutic management of patients with syndromes associating thrombotic manifestations and thrombocytopenia. The clinical suspicion for VITT-like syndromes should be considered in case of severe, otherwise unexplained, thrombotic events associated with thrombocytopenia in subjects without proximate heparin or adenovirus vector vaccine exposure. Immunoassays for the detection of anti-PF4/heparin antibodies certainly help in terms of timely recognition, but specific tests like HIPA and PIPA are indeed essential for a definitive diagnosis, which requires both clinical and laboratory collaborative networks. Therefore, the diagnostic and therapeutic challenge of anti-PF4 disorders is yet to be overcome. Both biomolecular research and epidemiological cohort studies are needed to address such issues and may improve the global management of patients with thrombotic thrombocytopenic syndromes.

## Data Availability

The original contributions presented in this study are included in the article. Further inquiries can be directed to the corresponding author.
